# Leptomeningeal Carcinomatosis Secondary to Adenocarcinoma of the Breast: A Cadaveric Case Report

**DOI:** 10.7759/cureus.12693

**Published:** 2021-01-14

**Authors:** Melissa Damaske, Victoria Panarese, Sean Casey, Madeline Feeney, Francis J Liuzzi

**Affiliations:** 1 Anatomy, Lake Erie College of Osteopathic Medicine, Bradenton, USA

**Keywords:** leptomeningeal carcinomatosis, breast cancer, brain metastasis

## Abstract

A rare complication of metastatic breast cancer, leptomeningeal carcinomatosis (LC) was discovered during routine cadaveric dissection of a 57-year-old Caucasian female who died from breast cancer metastasis to the brain. This pathology develops in only 5% of patients with metastatic breast cancer and presents with a number of neurological deficits. Progressive neurologic dysfunction is fatal, with a median survival of 10 to 15 weeks. In this case study, we examine the gross and microscopic features of LC and document the infiltration of metastatic cells into the brain parenchyma along the Virchow-Robin spaces.

## Introduction

Leptomeningeal carcinomatosis (LC) is the infiltration of the meninges by metastatic carcinoma of differing origins [1-2]. While only 2%-5% of breast cancer results in LC, the hematogenous spread of breast cancer to the leptomeninges is the most common cause of LC in adults [1-4]. This incidence is followed closely by lung cancer and melanoma [2-5]. LC presents an invasion of malignant cells into the arachnoid, the pia mater, and the subarachnoid space [3-4,6]. The cells may spread through the choroid plexus, vessels of the arachnoid, directly from the brain parenchyma, or along the cranial nerves to then invade the subarachnoid space [3,6]. Metastatic cell proliferation and accumulation in the subarachnoid space can cause obstruction of cerebrospinal fluid (CSF) flow and ventricular dilation. Patients who present with CSF flow obstruction have a poorer prognosis than those without obstruction [3,6].

## Case presentation

This case report involves a 57-year-old Caucasian female, whose cause of death was listed as metastatic breast cancer. Details of the subject’s clinical history, medical records, and initial presentation were not available because of the State of Florida Anatomical Board regulations.

**Figure 1:**
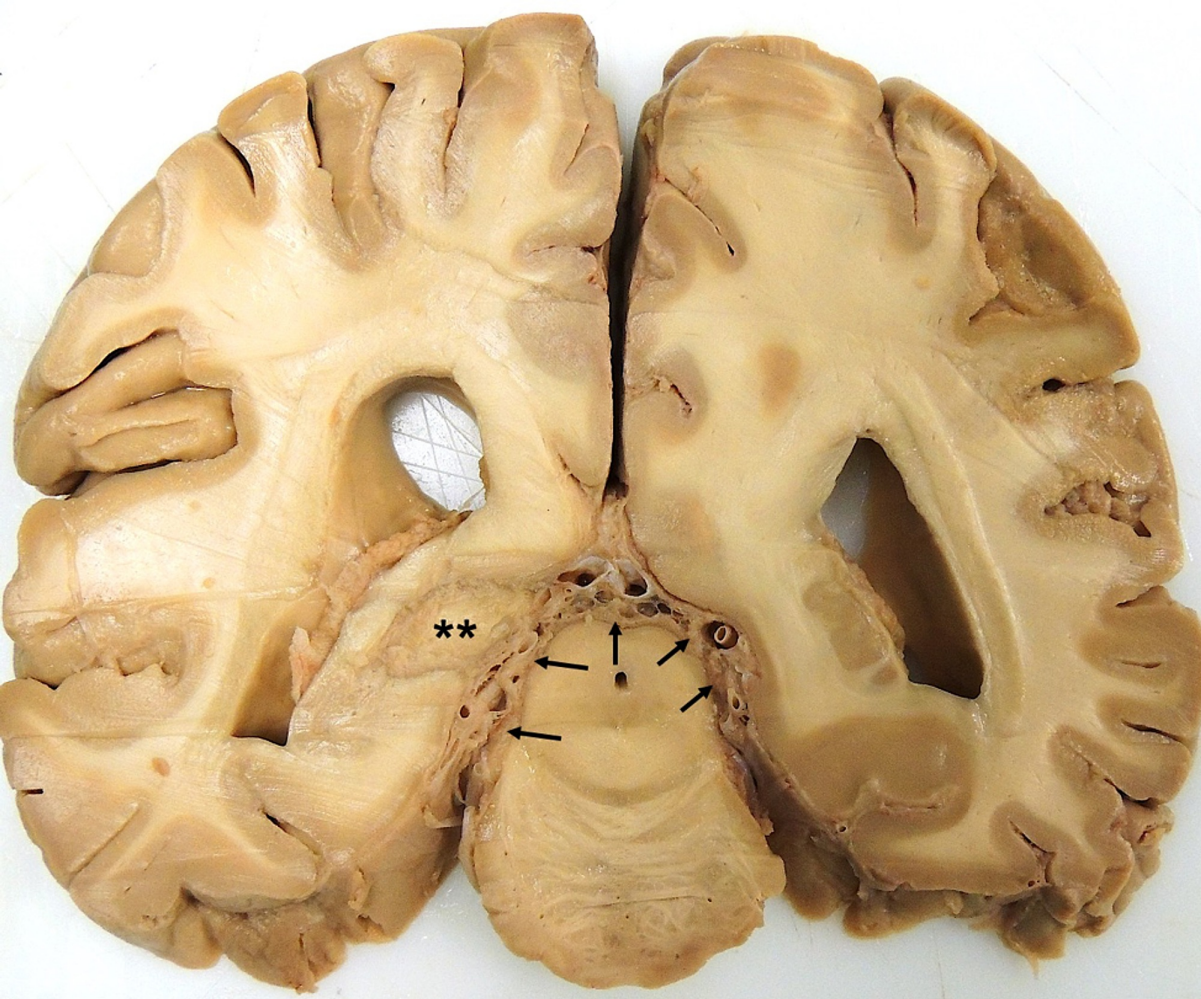
Coronal section showing metastatic infiltration of the meninges (arrows), an intraparenchymal mass (asterisks), and lateral ventricles that appear dilated

**Figure 2:**
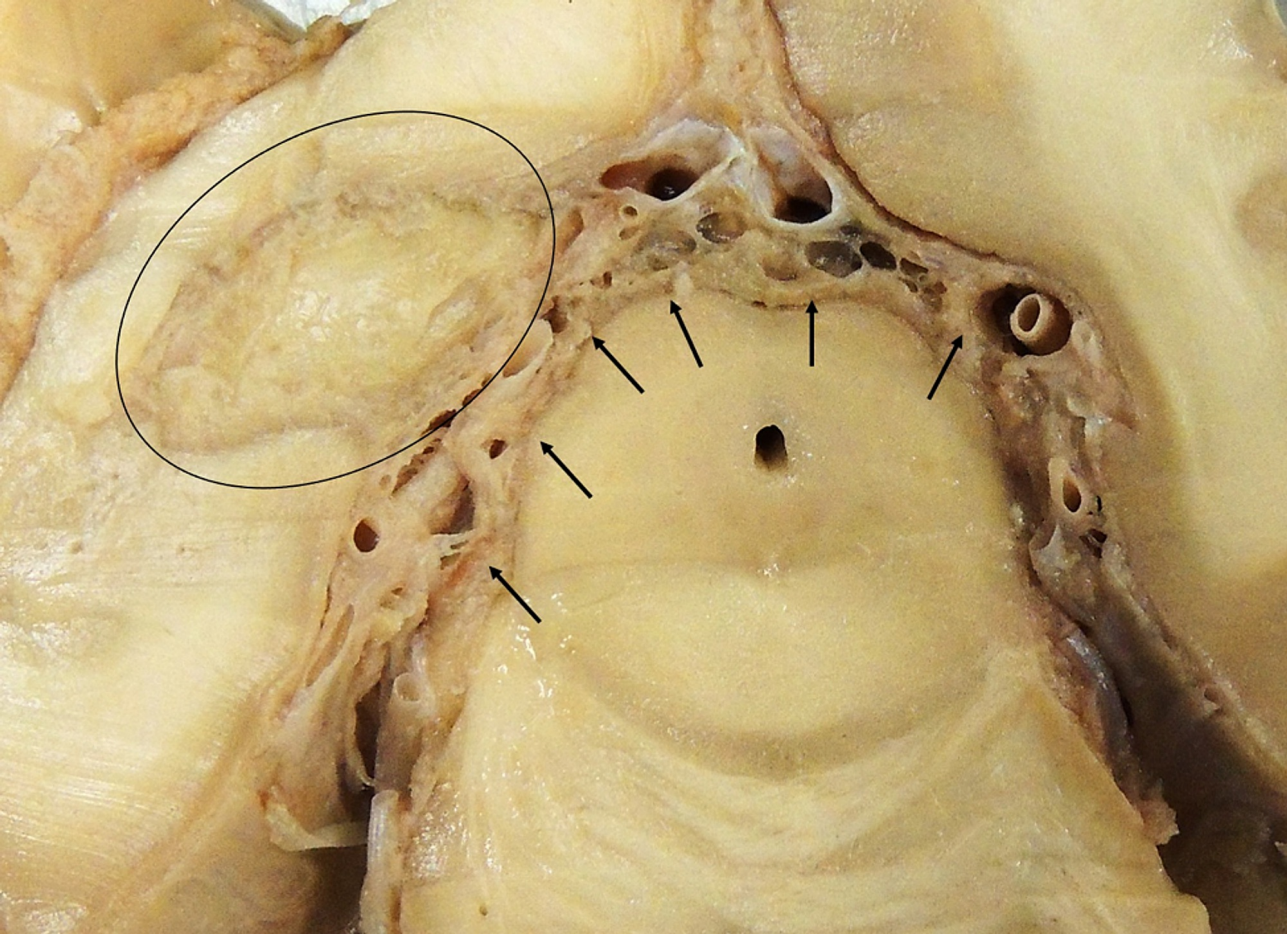
Meningeal infiltration (arrows) and intraparenchymal mass (oval)

The scalp was reflected, a craniotomy was performed, and the brain was removed. Two-centimeter coronal sections were made with a brain knife and examined for meningeal and parenchymal metastases. The sections revealed metastatic infiltration of the meninges between the cerebral hemispheres and the midbrain (Figure 1). In addition, a region of intraparenchymal infiltration was seen in the left hemisphere, extending approximately 1.5 cm into the hemisphere (Figure 1 and Figure 2). Both lateral ventricles appear dilated (Figure 1), suggesting obstruction of subarachnoid CSF flow.

An area of the region shown in the gross images was blocked out, placed in 4% formalin, and sent to Histowiz in Brooklyn, NY, for paraffin embedding, sectioning, and staining with hematoxylin and eosin (H and E).

**Figure 3:**
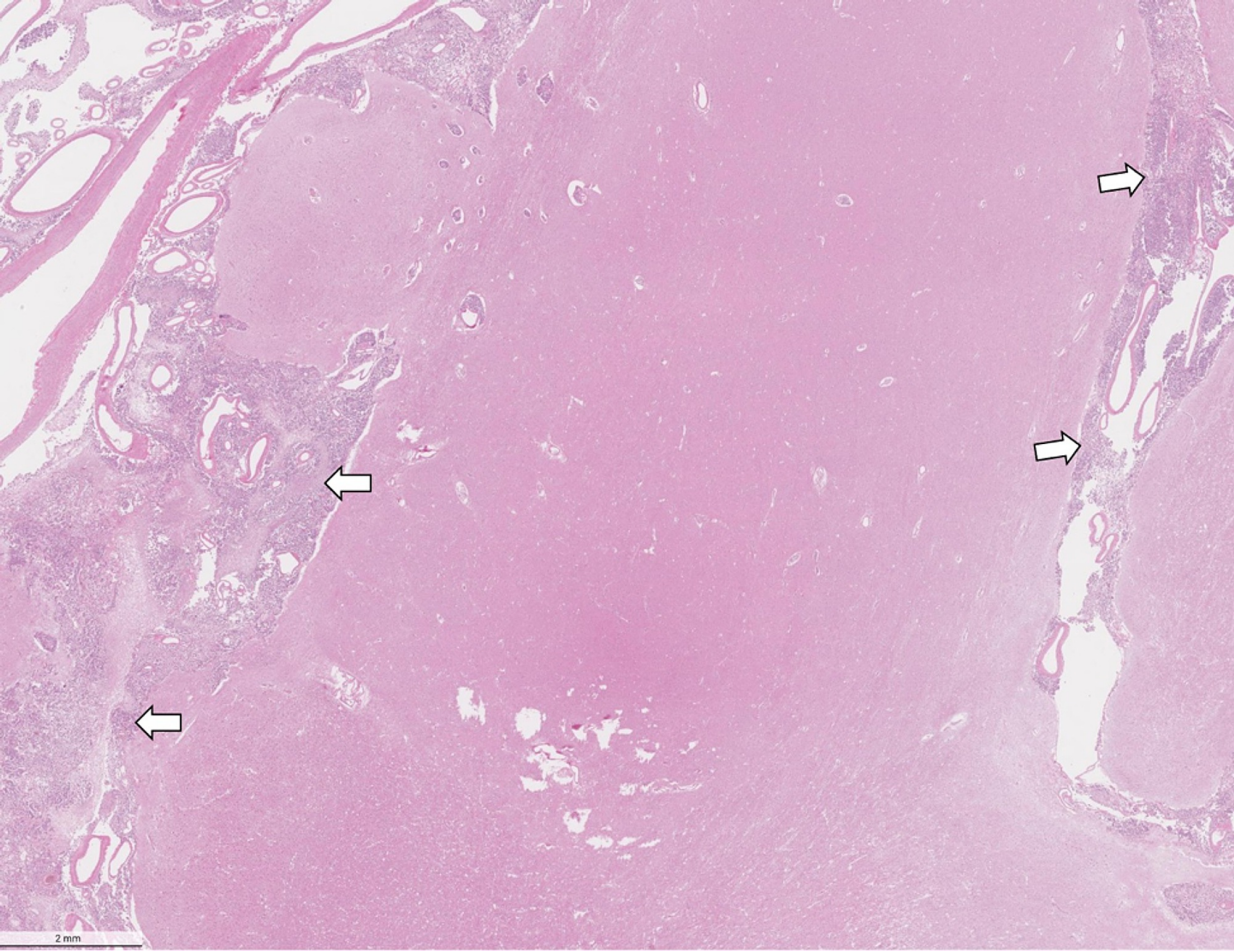
Low-power micrograph showing massive meningeal infiltration by metastatic cells (arrows)

**Figure 4:**
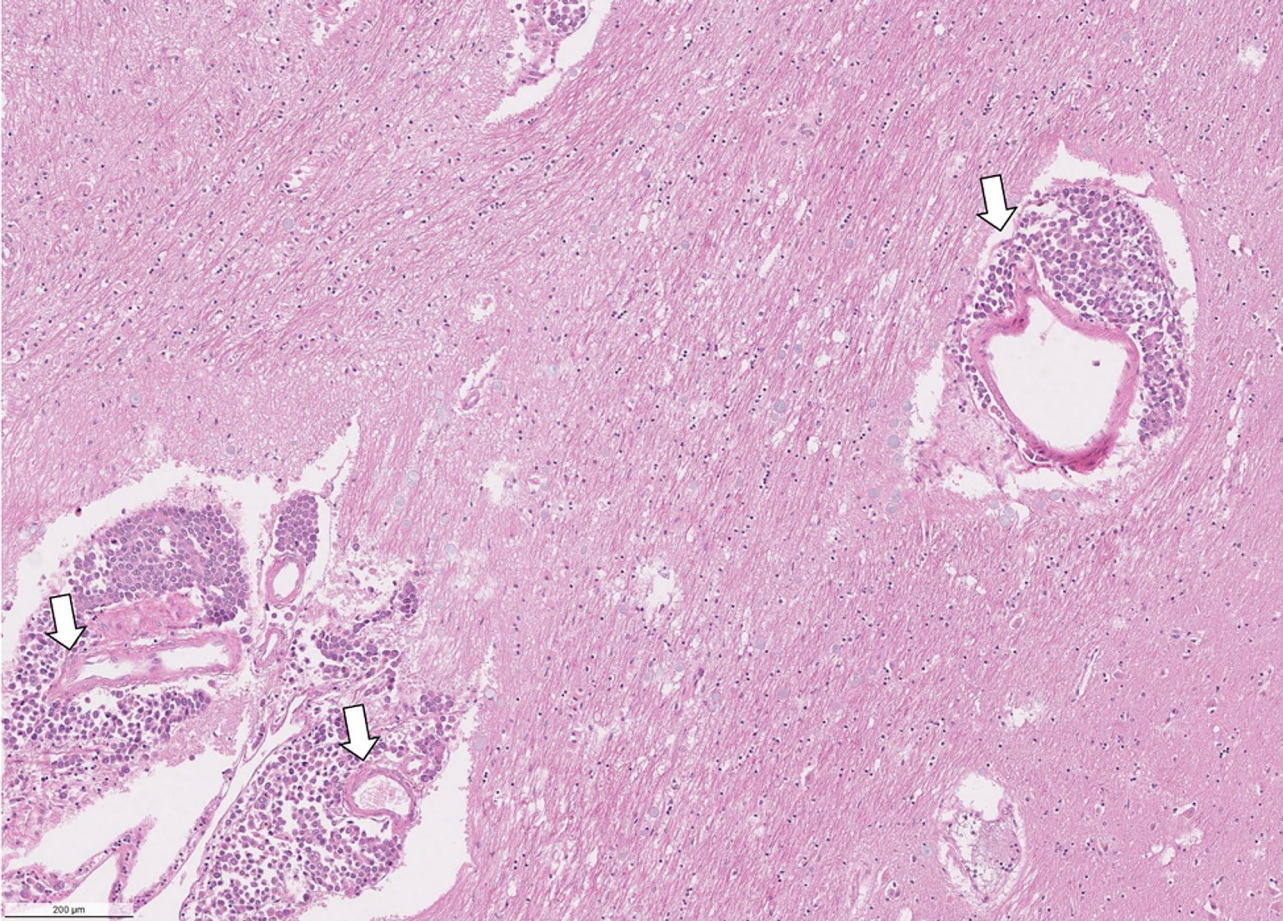
Higher-power micrograph showing perivascular metastatic cells that have migrated along the Virchow-Robin spaces (arrows)

**Figure 5:**
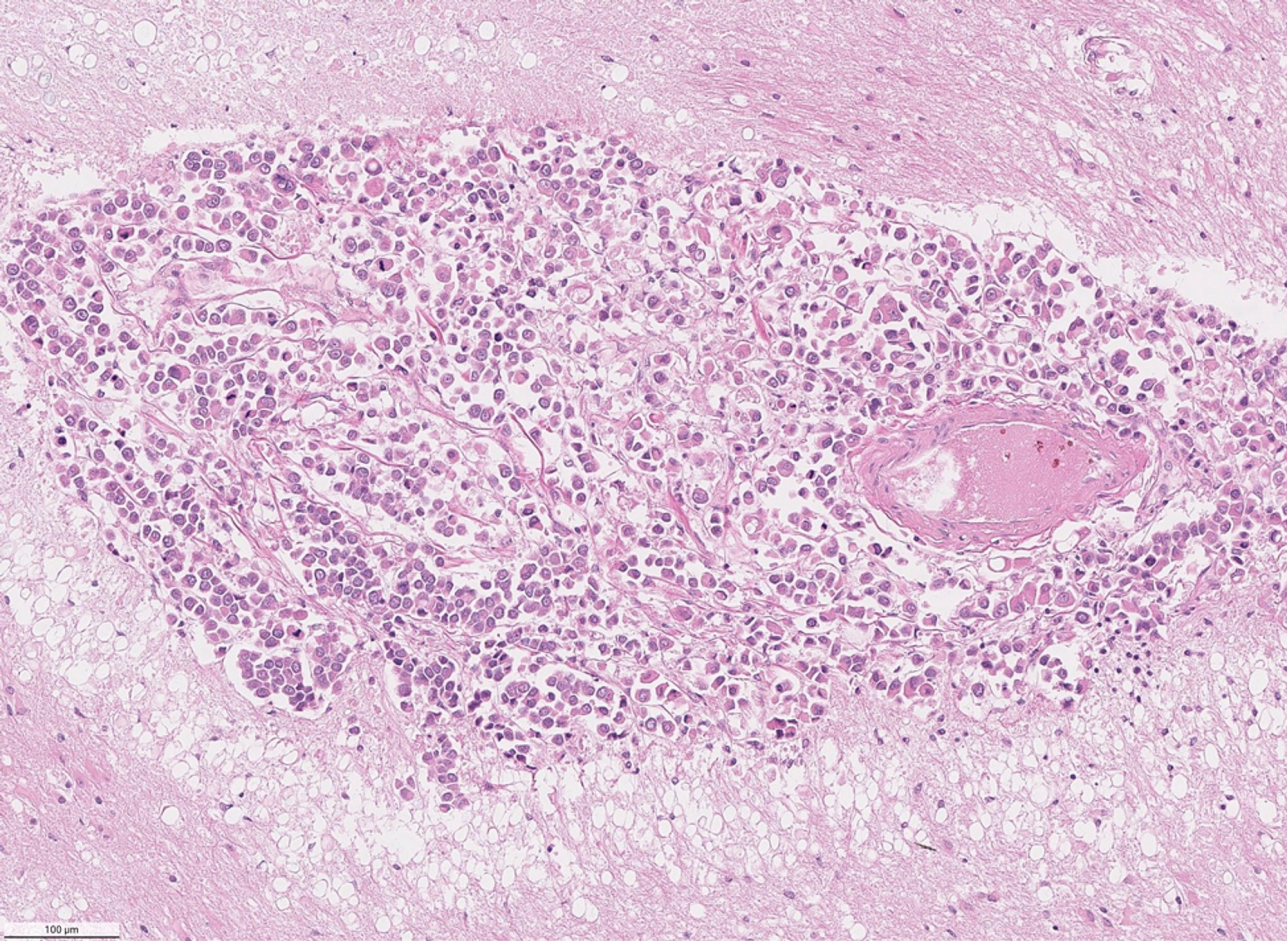
High-power micrograph showing a proliferative mass around an intraparenchymal blood vessel. Mitotic figures and cells with intracellular lumina are present.

**Figure 6:**
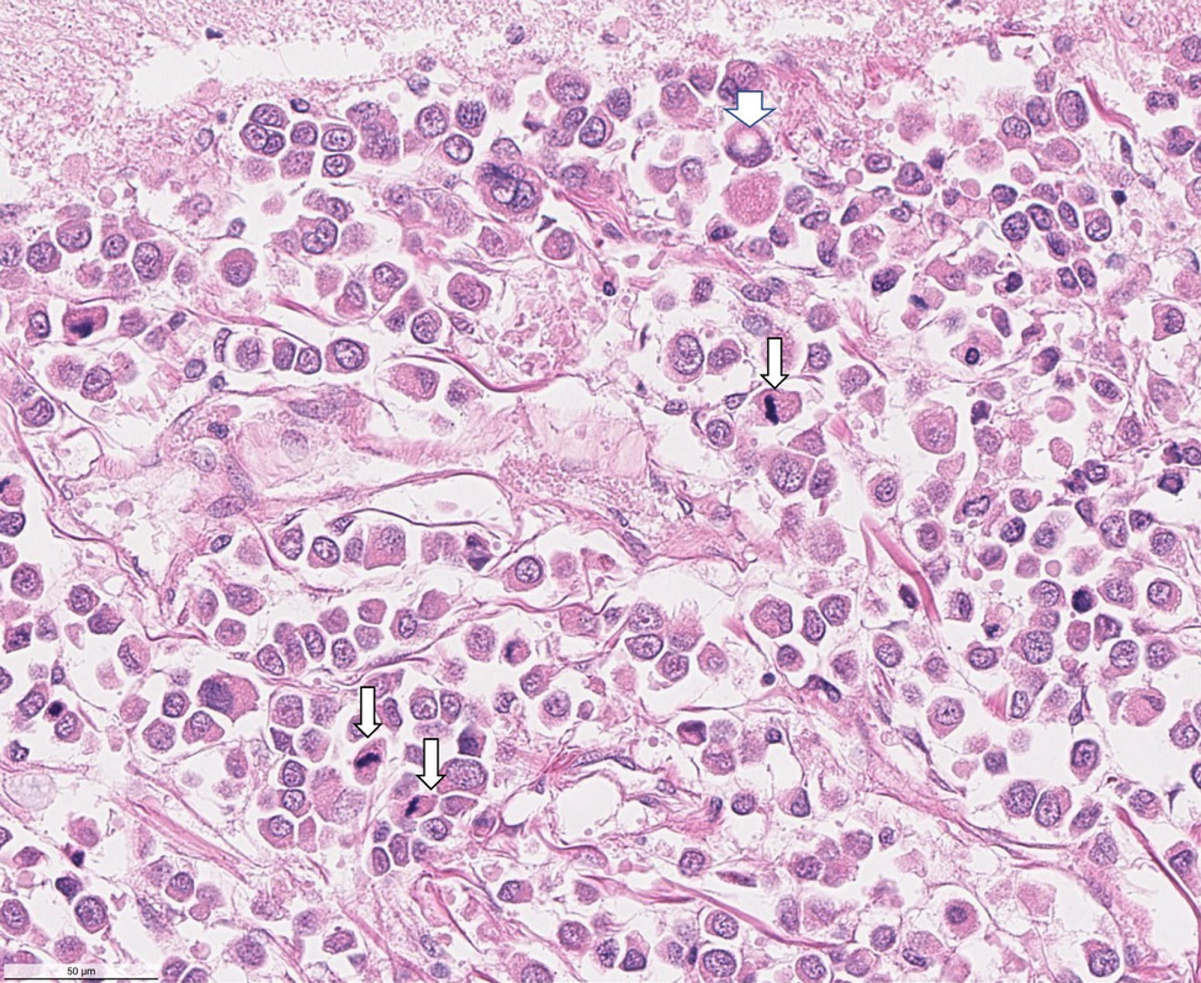
Higher-power micrograph showing mitotic figures (small arrows) and a signet cell with an intracellular lumen (large arrow)

Microscopic examination of the sections revealed extensive meningeal infiltration by metastatic cells, which encompassed branches of cerebral vessels within the subarachnoid space (Figure 3). There is perivascular tumor invasion around the branches of cerebral vessels within the subarachnoid space and migration of metastatic cells into the brain parenchyma along the Virchow-Robin spaces (Figure 4). In the brain parenchyma, there are numerous areas of intraparenchymal infiltration around blood vessels. These areas of infiltration exhibit cells with cytoplasmic pleomorphism, increased nuclear-to-cytoplasmic ratio, and intracellular lumina (Figure 5). Additionally, in these areas of the intraparenchymal tumor, there are numerous mitotic figures and signet cells with intracellular lumina (Figure 6).

## Discussion

Based on our postmortem observations of the gross brain and H&E-stained sections, along with the previous diagnosis of metastatic breast cancer, the most likely cause of death in this 57-year-old female was encephalopathy secondary to metastatic breast cancer. Our observations confirm a diagnosis of LC. Histological comparison of the brain metastases to the primary breast tumor was not possible in this case because the subject had undergone a bilateral mastectomy sometime before her death. If breast tissue was available, immunohistochemistry could have been performed.

LC develops in 5% of patients with metastatic breast cancer and presents with a number of neurological deficits [1-5]. Progressive neurologic dysfunction is fatal, with median survival ranging from 10 to 15 weeks [7]. Several treatment options are available to alleviate symptoms, although none offer a definitive cure. The extensive infiltration of tumor cells within the leptomeninges and subarachnoid space, along with the dilation of the lateral ventricles, suggests the likelihood of non-communicating hydrocephalus via obstruction of flow in the subarachnoid space. CSF flow is disrupted in around 30%-70% of cases of LC [3]. 

Symptoms of LC are typically non-specific and include headache, nausea and vomiting, and mental status changes [1,3-4,6]. With obstruction of CSF flow and increased intracranial pressure, symptoms may progress, resulting in vision changes, altered gait, seizures, and unconsciousness. Cranial nerve deficits presenting as diplopia may also occur [3-4,6].

The blood-brain barrier and blood-CSF barrier usually render the leptomeninges more resistant to metastases, which makes LC an uncommon occurrence. Theories of the pathogenesis of LC have implicated hematogenous, lymphatic, or direct spread from brain parenchyma [3-4,6]. Another theory suggests spread through the fenestrated endothelium of the choroid plexus, thus allowing the invasion into the CSF [3,6]. Such transendothelial migration of breast cancer cells may involve vascular endothelial growth factor (VEGF ) [4].

As of yet, LC remains to have curative treatment. The majority of patients who develop LC have already undergone chemotherapy (CTX) as per the standard protocols for breast cancer treatment [8]. The most common treatments include targeted radiotherapy (RT), whole-brain irradiation (WBRT), systemic and intrathecal chemotherapy [7]. However, systemic chemotherapy is limited by its ability to penetrate the blood-brain barrier [9]. One randomized controlled trial showed the efficacy and outcomes of intrathecal chemotherapy to provide no further benefit but to cause more harm as compared with systemic therapy [10]. This was based on the understanding of LC’s ability to penetrate and disrupt the blood-brain barrier and that systemic levels of chemotherapy were indeed able to penetrate and provide cytotoxic therapeutic levels [10].

Despite the array of therapies to treat solid breast tumors, the median survival rate of those with metastasis to the leptomeninges averages 12 weeks [6]. New research, however, has shown that craniospinal irradiation via helical tomotherapy (HT-CSI) has a better prognosis, although the goal is merely to alleviate the neurological symptoms and improve CSF flow [7]. Regarding chemosensitive breast cancers, treatment varies drastically by type. Seventy to 80% of invasive ductal carcinomas are estrogen-receptor (ER)-positive breast cancers, in which endocrine therapy consisting of aromatase inhibitors, tamoxifen, and fulvestrant has prolonged the overall survival from four months to 15 months [11]. In those cases that progressed to LC, overall survival increased from three months to seven months with the use of aromatase inhibitors [11]. When comparing subtypes of breast cancer, human epidermal growth factor receptor 2 (HER2)-positive breast cancer metastasizes to the brain more frequently [12]. Treatment guidelines depend on the number of brain metastases, with options being surgical resection, stereotactic radiosurgery, whole-brain radiotherapy, and a combination of systemic therapies, including but not limited to trastuzumab and tucatinib [12].

## Conclusions

LC is a rare complication of metastatic breast carcinoma. While it may originate from lung carcinoma or even melanoma, it most commonly results as a late manifestation of hematogenous metastasis of breast cancer. This case report presents a 56-year-old female who was diagnosed with breast adenocarcinoma; LC was incidentally found infiltrating the meninges between the cerebral hemispheres and the midbrain during cadaveric dissection. Histological analysis revealed cells with cytoplasmic pleomorphism, increased nuclear-to-cytoplasmic ratio, and intracellular lumina, as well as numerous mitotic figures and signet cells surrounding and infiltrating the vasculature. As of yet, there is no cure for LC, as systemic chemotherapeutic agents are unable to penetrate the blood-brain-barrier and the use of targeted radiation merely palliates symptoms of mass-effect.
